# Insider Threats to the Military Health System: A Systematic Background Check of TRICARE West Providers

**DOI:** 10.2196/52198

**Published:** 2024-04-09

**Authors:** David Bychkov

**Affiliations:** 1 UC Institute for Prediction Technology, University of California, Irvine, Orange, CA, United States

**Keywords:** TRICARE, health care fraud, Defense Health Agency, fraud, fraudulent, insurance, coverage, beneficiary, beneficiaries, background check, background checks, demographic, security clearance, FDA, Medicaid, Medicare, provider, provider referral, military, false claims act, HIPAA breach, OIG-LEIE, inspector general, misconduct, insider threat, information system, zero trust, data management, Food and Drug Administration, Health Insurance Portability and Accountability Act breach, Office of the Inspector General's List of Excluded Individuals and Entities

## Abstract

**Background:**

To address the pandemic, the Defense Health Agency (DHA) expanded its TRICARE civilian provider network by 30.1%. In 2022, the DHA Annual Report stated that TRICARE’s provider directories were only 80% accurate. Unlike Medicare, the DHA does not publicly reveal National Provider Identification (NPI) numbers. As a result, TRICARE’s 9.6 million beneficiaries lack the means to verify their doctor’s credentials. Since 2013, the Department of Health and Human Services’ (HHS) Office of Inspector General (OIG) has excluded 17,706 physicians and other providers from federal health programs due to billing fraud, neglect, drug-related convictions, and other offenses. These providers and their NPIs are included on the OIG’s List of Excluded Individuals and Entities (LEIE). Patients who receive care from excluded providers face higher risks of hospitalization and mortality.

**Objective:**

We sought to assess the extent to which TRICARE screens health care provider names on their referral website against criminal databases.

**Methods:**

Between January 1-31, 2023, we used TRICARE West’s provider directory to search for all providers within a 5-mile radius of 798 zip codes (38 per state, ≥10,000 residents each, randomly entered). We then copied and pasted all directory results’ first and last names, business names, addresses, phone numbers, fax numbers, degree types, practice specialties, and active or closed statuses into a CSV file. We cross-referenced the search results against US and state databases for medical and criminal misconduct, including the OIG-LEIE and General Services Administration’s (GSA) SAM.gov exclusion lists, the HHS Office of Civil Rights Health Insurance Portability and Accountability Act (HIPAA) breach reports, 15 available state Medicaid exclusion lists (state), the International Trade Administration’s Consolidated Screening List (CSL), 3 Food and Drug Administration (FDA) debarment lists, the Federal Bureau of Investigation’s (FBI) list of January 6 federal defendants, and the OIG-HHS list of fugitives (FUG).

**Results:**

Our provider search yielded 111,619 raw results; 54 zip codes contained no data. After removing 72,156 (64.65%) duplicate entries, closed offices, and non-TRICARE West locations, we identified 39,463 active provider names. Within this baseline sample group, there were 2398 (6.08%) total matches against all exclusion and sanction databases, including 2197 on the OIG-LEIE, 2311 on the GSA-SAM.gov list, 2 on the HIPAA list, 54 on the state Medicaid exclusion lists, 69 on the CSL, 3 on the FDA lists, 53 on the FBI list, and 10 on the FUG.

**Conclusions:**

TRICARE’s civilian provider roster merits further scrutiny by law enforcement. Following the National Institute of Standards and Technology 800, the DHA can mitigate privacy, safety, and security clearance threats by implementing an insider threat management model, robust enforcement of the False Claims Act, and mandatory security risk assessments. These are the views of the author, not the Department of Defense or the US government.

## Introduction

In response to the COVID-19 pandemic, the Military Health System expanded access to civilian providers. From 2020 to 2021, TRICARE, the Defense Health Agency (DHA) insurance scheme, maintained a steady beneficiary pool of 9.6 million military beneficiaries. Meanwhile, its civilian roster ballooned from 548,297 to 713,395 providers, a 30.11% increase over 1 fiscal year [1]. According to a report by the Department of Defense’s Office of the Inspector General (DODIG), the DHA’s Program Integrity (PI) office suspended medical record audits related to improper payments during the pandemic due to a lack of in-person investigators [2]. In 2021, the DHA-PI received 600 lead requests, opened 110 new cases, and managed 693 active cases. According to their annual reports, they sanctioned no additional health care providers since August 2020 [1,3]. Furthermore, only 80% of the provider directory information published by TRICARE’s managed care support contractors (MCSCs) was accurate [1]. Due to call center problems and technical challenges with the Defense Enrollment Eligibility Reporting System, the percentage of health care provider contracts compliant with TRICARE fluctuated between 79.5% and 94.1% throughout the first 47 months of the current T2017 contract [1]. Nevertheless, TRICARE promises beneficiaries that their affiliated civilian doctors meet “stringent quality and credentialing requirements” [1]. In the United States, National Provider Identification (NPI) numbers are the sole identifier for licensed clinicians. TRICARE does not publish NPIs in its provider directory. Military treatment facilities (MTFs), service members, and their families, therefore, lack a simple way to verify the bona fides of outside civilian providers.

Health care organizations may not purchase goods or services from excluded entities and vendors without jeopardizing their federal contracts [4,5]. The Centers for Medicare and Medicaid (CMS) require federally funded health care organizations to screen out providers against two sources at regular intervals: the Office of Inspector General’s (OIG) List of Excluded Individuals and Entities (LEIE) and the General Services Administration’s (GSA) SAM.gov exclusion list [6]. The OIG-LEIE is a comprehensive registry that excludes individuals and entities from participating in federally funded health care programs for a range of reasons, including patient abuse or neglect, billing fraud, and drug-related convictions [7]. According to Burton et al’s [8] demographic analysis of 1289 physicians on the OIG-LEIE between January 2008 and December 2013, a total of 509 were excluded for license revocation or suspension (Social Security Act 1128 (b)(4)), 280 for Medicare/Medicaid fraud conviction (Social Security Act 1128 (a)(1)), 123 for another type of health fraud conviction (Social Security Act 1128 (a)(1)), and 191 for felony controlled substance disbursement (Social Security Act 1128 (a)(4)) [8]. Male physicians represented nearly 85% of the total excluded physicians but accounted for nearly 70% of the general physician population [8]. One long-term care facility was fined US $376,000 for multiple violations of exclusion rules, with fines typically exceeding tens of thousands of dollars per violation [9]. In FY2022, the Department of Health and Human Services’ (HHS) OIG Medicaid Fraud Control Unit reported 10,604 and 7202 open criminal and civil investigations, respectively [10].

The GSA-SAM.gov is a list of corporations forbidden from doing business with the US government. In April 2003, the HHS debarred a medical supplies company for 5 years after the owner pleaded guilty to Medicare fraud [11]. As HHS did not debar the individual’s company, he transferred ownership of the company to his wife and they continued the scheme. After investigators discovered the corporate change, the couple transferred the company ownership again to a neighbor. Two years later, the neighbor sold the company back to the original owner’s wife. To prevent further discovery, the wife changed her last name to her maiden name. Thanks to these tactics, the couple managed to defraud federal health programs for the entire 5 years of debarment. Under procedures outlined in Federal Acquisition Regulation (FAR) subpart 9.4.19, an agency suspending and debarring official may suspend any contractor upon receiving an allegation that a contractor is not acting responsibly [12]. The suspension is enacted by listing the contractor in the excluded status on the GSA-SAM.gov list and notifying the contractor in writing. Under 48 C.F.R. § 9.405, no award can be issued to a contractor suspended, proposed for debarment, debarred, or otherwise award ineligible unless the agency head or designee determines in writing that a “compelling reason” exists [5]. For the DHA to conduct business with an excluded health provider, as per 10 U.S.C. § 2393, the Secretary of Defense must provide the GSA notice of the “Compelling Reason Determination Pursuant to 48 C.F.R. § 9.405” for publication on the web [13]. Thirty agencies contribute data to the Interagency Suspension and Debarment Committee (ISDC) and the GSA-SAM.gov list. As of the time of publication, the ISDC has published no compelling reason determinations associated with TRICARE’s civilian provider network [14].

Direct or indirect federal reimbursement for goods or services rendered by an excluded individual or entity is prohibited by the False Claims Act, FAR 9.404 “Exclusions in the System for Award Management’' and the Civil Monetary Penalties Law [15]. This includes reimbursement for salaries, benefits, or items claimed or billed by licensed health care providers and administrative personnel. Hospitals, equipment suppliers, drug manufacturers, and health management organizations that serve federal programs must use the OIG-LEIE and GSA-SAM.gov to screen out inappropriate employees and contractors [16]. Billing federal health care programs for services rendered by excluded providers can result in a minimum penalty of US $10,000 per instance [17]. To automate this process, McKesson, a revenue cycle management and electronic health record software provider has integrated exclusion monitoring tools into their products [18]. As TRICARE still accepts billing claims by fax and mail, fraudsters can potentially thwart automated exclusion screening processes [19].

The DHA provides no mandatory information security training to outside contractors. Although the Health Insurance Portability and Accountability Act (HIPAA) of 1996 requires health care providers to perform security risk assessments (SRAs) [20], 17% of respondents to the 2021 Healthcare Information and Management Systems Society Healthcare Cybersecurity Survey reported not having a budget for risk assessments [21]. In the same survey, 83% of respondents had experienced a cyberattack. Due to budget or logistical concerns, 26% had reduced their overall cybersecurity budget [21]. Continuous personnel screening is an effective mitigation practice against insider threats. Malicious nonstate actors seek negligent insiders to help them target personal identifying information (PII) and personal health information (PHI) contained in electronic health records at medical practices [22].

Approximately 18% of all service members receive security clearances [23]. Service members need to discuss their medical conditions with health providers without fear of data breaches or blackmail by an adversary [24]. Military personnel have a reasonable expectation that TRICARE-credentialed health care providers are not fugitives from justice, in violation of international sanctions, a threat to national security, or associated with a cyber breach. Unfortunately, military-affiliated consumers are 76% more likely than other adults to experience medical benefit fraud and identity theft [25]. Nicholas et al [26] performed a cross-sectional study of 8204 Medicare beneficiaries who received care from excluded providers. They revealed that patients treated by fraudsters experienced a 13%-23% increased risk of mortality and an 11%-30% higher risk of hospitalization.

In addition to the OIG-LEIE and GSA-SAM.gov, multiple public databases exist to search names concerning each of these issues, including:

- HHS’ Office of Civil Rights’ HIPAA Breach Report affecting 500 or more patients (HIPAA) [27];
- HHS-OIG list of fugitives (FUG) wanted for health care fraud, abuse, or child support obligations [28];
- International Trade Administration’s Consolidated Screening List (CSL) of parties for which the US government maintains restrictions on exports, reexports, or transfers of items [29];
- The Federal Bureau of Investigation’s (FBI) list of January 6th capitol breach defendants [22,30];
- Lists of providers excluded by 15 of 21 state Medicaid programs for fraud, neglect, and abuse (state) [31];
- The US Food and Drug Administration’s (FDA) debarment lists for illegal drug imports, food imports, and drug product activity [32]; and
- DHA’s Sanction List for TRICARE-specific billing fraud and patient abuse (DHA sanctions) [33].

This study, therefore, aims to determine if TRICARE refers its beneficiaries to providers found on government exclusion or sanction lists. If so, we aim to identify their professional and geographic characteristics. Finally, we offer recommendations to mitigate potential threats posed by excluded providers to the safety, privacy, and security clearances of service members.

## Methods

### Overview of the Study Area

Time constraints imposed by the 1-year time limit of our Human Research Protection Program required us to choose and investigate only one MCSC’s provider roster. TRICARE’s two main MCSCs, Health Net Federal Services, LLC (TRICARE West) and Humana Inc (TRICARE East) [1], receive approximately US $7.2 billion and US $7.87 billion per year, respectively, to ensure military medical readiness in their respective regions [34]. Additional MCSCs provide services to niche beneficiary populations, including Johns Hopkins Medicine (US Family Health Plan) and International SOS Government Services, Inc (TRICARE Overseas) [1]. These MCSCs do not display the NPIs of their health care providers on the web. From this group, we chose TRICARE West randomly.

TRICARE West is currently operated by Health Net Federal Services, LLC across 21 states: Alaska, Arizona, California, Colorado, Hawaii, Idaho, Iowa (excluding the Rock Island Arsenal area), Kansas, Minnesota, Missouri (excluding the St. Louis area), Montana, Nebraska, Nevada, New Mexico, North Dakota, Oregon, South Dakota, Texas (Amarillo, Lubbock, and El Paso areas only), Utah, Washington, and Wyoming.

### Providers

To gather further information on health care providers in the search area, we evaluated national and statewide trends from the Health Research Services Administration’s (HRSA) National Practitioner Data Bank related to licensure, adverse events, malpractice, Drug Enforcement Administration enforcement, and exclusions [35]. HRSA also publishes zip code–level data on health provider shortage areas (HPSAs) [36] and the availability of nearby Federally Qualified Health Centers (FQHCs). FQHCs are nonprofit medical facilities that provide primary care to an area or people in need, offer a sliding fee scale, provide complete services, have a quality assurance program, and maintain a governing board of directors [37]. For rural communities where TRICARE providers refuse new patients or lack available appointment times, FQHCs may be an excellent closer option than an MTF. They compare favorably to private providers for patient access, safety, and satisfaction [38]. Due to their nonprofit mission, however, many FQHCs lack digital health care IT resources [39]. No studies have investigated TRICARE beneficiary use of FQHCs.

### The Patient Population

TRICARE West’s 3.7 million patients reside primarily in Texas, California, Washington, Colorado, and Arizona [40]. The Defense Manpower Data Center provides some details on the distribution of military service members and their families. For example, Texas is home to the highest concentration of service members from both the Army (16.9%) and Air Force (12.9%). California houses the largest number of active duty Marines (36.6%) [41]. Most active Space Force Guardians (24.7%), on the other hand, are based in Colorado [41]. By contrast, the largest concentration of Navy active duty members reside in Virginia (26.5%) [42]. Although one-third of service members move every year, no public-facing data indicate how many TRICARE West beneficiaries transition to TRICARE East or vice versa [43]. While service members are somewhat healthier than civilians, today’s TRICARE providers must treat the same conditions that impact the rest of society, such as cardio- and neurovascular diseases, sexually transmitted infections, substance use disorders, metabolic disorders, and mental health issues [44-46]. According to the Medical Expenditure Panel Survey, a nationwide questionnaire, TRICARE-covered families nationwide reported inferior access to medical care when compared to uninsured, commercially insured, and privately insured peers [47]. Furthermore, military families dealing with complex pediatric care reported worse outcomes than civilians [47]. Approximately 40% of military families have children [48]. At least 1 in 12 rely on Medicaid to provide supplemental coverage for their children [49].

### Data Sources

TRICARE West publishes the first and last names, specialty type, practice type, company names, and contact information of TRICARE-credentialed civilian providers on their public-facing provider directory, which was accessed on the TRICARE West website [50] for this study.

Between January 1-31, 2023, we used TRICARE West’s provider directory to search for all names within a 5-mile radius of 798 United States Postal Service–designated zip codes within TRICARE West’s territory of 12,574 zip codes. We copied and pasted all raw results into an Excel (.xls) file (Microsoft Corporation).

To ensure compliance with the terms and conditions of the TRICARE provider directory, all data were manually accessed.

### Zip Code Search Selection

To ensure each batch of results included the largest possible number of TRICARE provider names, we limited the scope of each search query on the TRICARE West provider directory to those zip codes known to contain at least 10,000 residents and 1 credentialed medical provider. Population estimates were gathered from the 2020 US Census [51]. For each state, 38 zip codes were selected for searching on the TRICARE West provider directory. These 798 zip code searches represent 6.1% of TRICARE West’s total land coverage area.

### Provider Search Parameters

The TRICARE West provider directory displays first, last, and business names; full addresses; phone and fax numbers; degree type; provider gender; specialty; and active/closed status.

### Analysis Parameters

To evaluate the data, we gathered the most current .xls versions of the OIG-LEIE and GSA-SAM.gov, the HIPAA list, state lists, CSL, FDA lists, the FBI list, the DHA Sanctions List, and FUG. Using the VLOOKUP function in Excel, we cross-referenced the providers’ first, last, and corporate names (with and without zip code qualifiers) against each exclusion, sanction, and violation list. VLOOKUP search strings are useful database tools for detecting fraud patterns in spreadsheets, including common names or locations [52,53].

### Ethical Considerations

This study relied on no confidential data. It was conducted with an exemption from the Human Resource Protection Program of Defense Acquisition University, received on January 30, 2023.

## Results

### Overview

Our search of the TRICARE West provider directory yielded 111,619 raw results across the 798 zip code areas. Searches of 54 zip codes yielded no entries, including 21 searches in Missouri, 18 in Wyoming, 5 in Iowa, 5 in Alaska, 4 in Washington, and 1 in Texas.

After filtering out 72,156 (64.65%) entries for duplicates, closed offices, and data located in external states, we established our baseline list of 39,463 TRICARE West active provider names. This group accounts for 5.53% of TRICARE’s 2021-2022 nationwide civilian roster [1].

**Figure 1.**
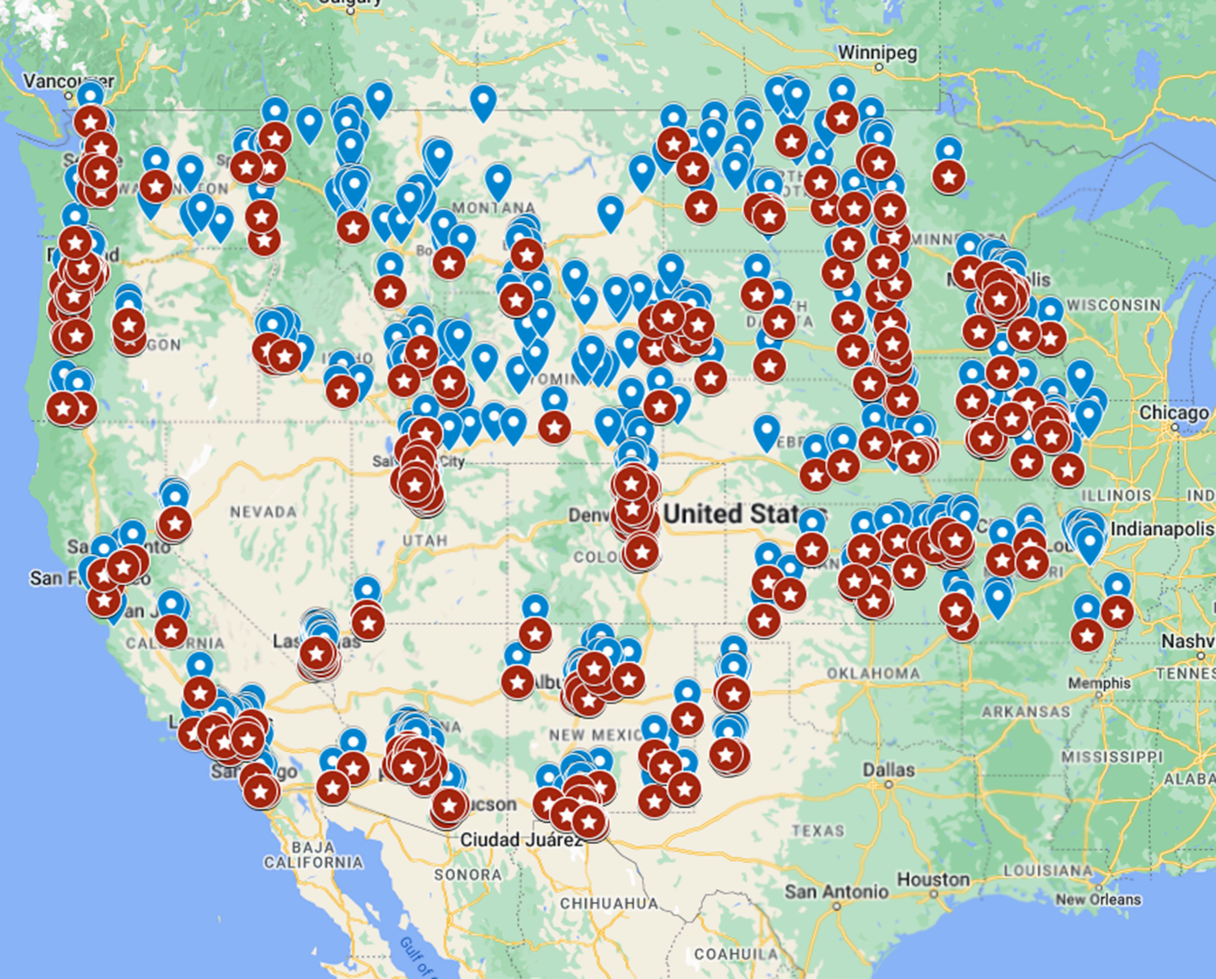
Map of search area zip codes (in blue) vs zip codes where provider names were matched against exclusion lists (in red). Map data ©2024 Google, INEGI.

Within the baseline group, 2398 (6.08%) provider names matched the first and last names of individuals and business owners found on 10 federal and state regulatory watch lists (Figure 1).

### Exclusion Types

Among the 2398 names, 2197 appear on the OIG-LEIE and 2311 appear on the GSA-SAM.gov. Within the group, 2 appear on the HIPAA list, 69 appear on the CSL list, 10 appear on the FUG, 15 appear on the FDA lists, 53 appear on the FBI list, and 54 appear on 15 different state Medicaid exclusion lists (Alaska: 0; California: 38; Hawaii: 0; Idaho: 0; Iowa: 1; Kansas: 0; Minnesota: 0; Missouri: 0; Montana: 0; North Dakota: 0; Nebraska: 0; Nevada: 1; Texas: 20; Washington: 0; Wyoming: 0).

Our search matched 1997 providers with 2 total exclusion types, 230 providers with 1 type, 158 providers with 3 exclusion types, 12 providers with 4 exclusion types, and 1 provider with 5 exclusion types. All providers with 2 or more exclusions have their first, last, or corporate names appear on the OIG-LEIE and GSA-SAM.gov exclusion lists. All names that appear on the FDA lists, HIPAA list, FUG, and CSL also appear on the OIG-LEIE or GSA-SAM.gov. Most providers with names on the FBI list (50/54) appear on the GSA-SAM.gov.

One name matched 1 provider on the DHA’s Sanctioned Provider List for TRICARE-specific offenses. To protect the provider’s privacy, we will not identify their state or other matching characteristics. Since 1990, the DHA Sanctions List has included no new providers in Utah or Minnesota (Table 1).

**Table 1.** Exclusion search vs actual exclusion enforcement by TRICARE.^a^

Top 5 states for exclusion-provider name matches	State TRICARE population, n	Historical enforcement by TRICARE, n	Exclusions found for TRICARE West providers, n
Utah	80,390	0	264
Minnesota	72,931	0	227
Kansas	120,503	6	212
Colorado	253,214	1	201
Washington	348,694	3	180
Total	N/A^b^	10	1084

a Since 1990, the Defense Health Agency has sanctioned 6 providers in Kansas, 1 in Colorado, and 3 in Washington State. They sanctioned no providers in Utah or Minnesota, the states where we found the highest concentration of names with exclusions.

b N/A: not applicable.

### Provider Characteristics

Provider names linked to exclusions were also associated with a medical degree (doctor of medicine [MD] n=1288, 54%; doctor of osteopathic medicine [DO] n=199, 8%). Family medicine was the top specialty (n=324, 13.5%), followed by nurse practitioner (n=148, 6.2%), internal medicine (n=112, 4.7%), optometrist (n=99, 4.1%), and pediatrics (n=91, 3.8%). Diploma types with the fewest exclusions included master of nursing (MN; n=1), registered behavior technician (RBT; n=1), licensed clinical psychologist (LCP; n=1), physical therapist (PT; n=1), and certified registered nurse anesthetist (CRNA; n=1). Our results include information about provider specialty and diploma type. Our results also included information on provider gender. Male providers accounted for 59.42% of the exclusions, while female providers accounted for 40%. Two providers did not report their gender.

### Provider Location

States with the highest concentrations of names associated with exclusions within the TRICARE West network were Utah (11%) and Minnesota (9%). The five zip codes with the highest exclusions were 84096 (Herriman, UT; n=18), 84062 (Pleasant Grove, UT; n=17), 99669 (Soldotna, AK; n=16), 84790 (Washington County, UT; n=14), and 80524 (Fort Collins, CO; n=14). The five zip codes with only 1 total exclusion were 96782 (Honolulu, HI; n=1), 79159 (Amarillo, TX; n=1), 84003 (Utah County, UT; n=1), 83401 (Bonneville County, ID; n=1), and 99505 (Anchorage, AK; n=1).

Finally, we conducted a follow-up TRICARE West directory search of how many MTFs operate within a 100-mile radius of those zip codes with the highest concentration of excluded provider name matches (Table 2). Whereas 96782 (Honolulu, HI) had 9 MTFs within a 100-mile radius and 1 provider associated with an exclusion, 83401 (Bonneville County, ID), 84790 (Washington County, UT), and 79159 (Amarillo, TX) have no MTF alternatives to provider names associated with exclusions.

**Table 2.** Top 10 zip codes for provider names matched to exclusions.

Top 10 zip codes	Total names, n	MTFs^a^ within 100 miles, n
84096 (Herriman, UT)	18	2
*84062 (Pleasant Grove, UT)* ^*b*^	17	2
*99669 (Soldotna, AK)*	16	2
*84790 (Washington County, UT)*	14	0
*80524 (Fort Collins, CO)*	14	2
96782 (Honolulu, HI)	1	9
*79159 (Amarillo, TX)*	1	0
*84003 (Utah County, UT)*	1	2
*83401 (Bonneville County, ID )*	1	0
*99505 (Anchorage, AK)*	1	2

a MTF: military treatment facility.

b Italicized zip codes are federally designated health provider shortage areas.

## Discussion

### Significance of Findings

This is the first academic study to assess the level of compliance of a federal health care program’s provider directory with criminal background check laws. In addition to evaluating provider names on databases for medical fraud, we expanded our search to include domestic terrorism, financial crimes, child support delinquency, and data breaches. Our matches included 28 fugitives from justice and 58 January 6th defendants.

Our results align with historical trends contained in Total Force Medical Readiness (TFMR) reports [54]. Between 2013 and 2021, overall individual medical readiness among nondeployed military components dropped 6 points [1]. In Q4 2013, 75% of the total force reported being “fully medically ready” (ie, satisfactory dental health, completion of periodic health assessments, deployment-limiting medical conditions status, current immunization status, completion of medical readiness lab tests, and possession of required individual medical equipment). By Q4 FY2021, readiness dropped to 69% [1]. Currently, TFMR reports do not indicate if they rely on data providers who received improper payments. Furthermore, they display no margin of error.

Our demographic findings are consistent with recent and historical reviews of excluded providers. In a cross-sectional study on physician exclusions from 2007 to 2017, Chen et al [55] reported that the total number of physician exclusions grew by 20% to include nearly 0.3% of all US physicians. Exclusions are more common in the West and Southeast among male physicians. In line with Burton et al’s [8] demographic analysis of the OIG-LEIE the majority of the provider names we sampled from TRICARE West’s provider directory have specialty training in family medicine and appear on the GSA-SAM.gov.

Geospatial data may help investigators link medical fraud and adverse events. According to HRSA’s database of HPSAs, 8 of the top 10 zip codes identified by our study for provider names with exclusions lack primary care workers [36]. Indeed, Zhang et al [56] forecasted that Western states will face an acute shortage of 69 physicians per 100,000 residents by 2030.

During the pandemic, HRSAs reported an increase in the number of adverse action reports and medical malpractice payments within the TRICARE West states with the most provider name-exclusion matches (Table 1). The most common adverse event is a patient fall [57]. Increased patient falls at hospitals and nursing homes are typically caused by breakdowns in clinical communications, including systemwide failures in teamwork and failures to consistently follow policies [58]. In Utah, for example, there were 359 adverse event reports in 2019—the highest number recorded in the state’s history [59]. In 2013, there were 555 adverse events in Colorado. By 2020, they peaked at 1215 [57].

We matched the most provider names against exclusions in states where the DHA sanctioned few or no physicians. For example, we detected the highest total number of potentially excluded providers in Utah (n=264), Minnesota (n=227), Kansas (n=212), Colorado (n=201), and Washington (n=180; Table 1). We found the lowest number of exclusion-provider name matches in Idaho (n=32), Missouri (n=21), Nebraska (n=15), and Montana (n=14). The TRICARE West states with the lowest number of DHA-sanctioned providers since 1990 were Utah (n=0), Minnesota (n=0), Colorado (n=1), Oregan (n=0), Montana (n=1), North Dakota (n=0), South Dakota (n=0), and Washington (n=3). Whereas the OIG-LEIE contains a total of 77,621 providers banned from federal health programs, the DHA Sanctions List contains only 129 provider names. In other words, medical fraudsters may have evaded detection by operating in states where the DHA only checks provider names against their own sanctions list.

TRICARE’s financial statement reveals opportunities for increased enforcement of the False Claims Act. The 2022 DHA Annual Report shows US $900,000 collected from excluded providers in 2020 and US $100,000 in 2019—down from a high of US $1.4 million in 2017 [1]. In their FY2021 evaluation of the TRICARE Program, the DHA reported an average annual expenditure of US $2251 on the typical single beneficiary [1]. If 120 (5%) of the 2398 identified TRICARE West providers billed for services for 3 beneficiaries over the preceding calendar year, the DHA could recoup at least US $810,360. Under the False Claims Act, the agency could require each of those providers to pay an additional US $10,000 per patient in penalties (ie, US $3,600,000 in penalties; US $4.4 million in total improper payment recoveries) [60].

### Recommendations

Service members and their families need tools to protect themselves from identity theft and medical fraud. HIPAA requires health care providers, including TRICARE West, to safeguard PHI [61]. HIPAA requires health care organizations to conduct regular SRAs to evaluate external and insider threats to PHI and other sensitive data. In addition to their appearance on the OIG-LEIE, two provider names on TRICARE West’s roster were associated with data breaches impacting over 500 medical patients. At least 450,000 TRICARE beneficiaries have an active security clearance [62]. TRICARE has not published a data breach disclosure since 4.9 million patient records disappeared in 2011 [63].

All federal agencies must implement a zero trust (ZT) architecture by 2027. The zero trust paradigm of information security requires participants to operate on a “need to know” basis. We recommend that the DHA prioritize the deployment of a ZT-based Insider Threat Management Model to protect the PHI of service members with security clearance (Table 3). Our model continuously screens the DHA’s civilian providers against federal and state databases. Aligned with the National Institute of Standards and Technology (NIST) 800-207 [64] and the CISA Zero Trust Maturity Model [65], it helps TRICARE administrators wall off providers linked to exclusions from beneficiaries and their data. Effectively, TRICARE patients with confidential-level security clearance or higher gain access to a filtered roster of health care providers. If a search of TRICARE’s website by a National Security Agency engineer with a Top Secret or Sensitive Compartmented Information clearance in Maryland for “John + Smith” matches the name of an excluded provider on the OIG-LEIE and GSA-SAM.gov in a beneficiary’s state, the roster would only display licensed, bona fide physicians. If none exist nearby, the roster would display the closest MTF. If no MTFs exist within a 100-mile radius, TRICARE’s website could display nonemergency medical transportation options to the nearest MTF, secure telehealth options, and covered care options at the nearest out-of-network screened provider. According to our Insider Threat Management Model, any providers in the TRICARE network associated with 3 or more exclusions would lose all access to *any* beneficiaries with clearance. Those beneficiaries need to be transferred to the nearest MTF. Patients who receive care from providers with 3 or more exclusions face higher risks of adverse events [9].

**Table 3.** Insider Threat Management Model. The system continuously vets all civilian network providers.

Beneficiary security clearance level	Doctor’s exclusions		
	1 exclusion	2 exclusions	≥3 exclusions
Confidential	Yellow	Yellow	Red
Secret	Yellow	Orange	Red
Top Secret	Yellow	Red	Red
Sensitive	Yellow	Red	Red
Sensitive Compartmented Information	Yellow	Red	Red

HIPAA entitles TRICARE beneficiaries to specific tools for medical privacy. For example, they may opt out of the Joint Health Information Exchange (JHIE), an electronic platform for transmitting medical data to civilian providers [7]. Currently, the JHIE opt-out system requires a paper-pencil request. As an alternative, the DHA could offer an electronic JHIE opt-out button in current Defense Finance and Accounting Service (DFAS) myPAY dashboards. DFAS myPAY offers multifactor authentication and paperless transactions to mitigate the threat of lateral nonauthorized movement of PII and PHI beyond the control of the beneficiary [66,67]. Our Insider Threat Management Model complements rather than replaces current federal agency mandates for periodic security training of all DHA employees, contractors, and credentialed providers; implementation of strict password, SRAs, and account management practices; explicit security agreements and access restrictions; PHI, PII, and other sensitive information only made available to those who require it; and use of a security information and event management [68] solution. Our model aligns with the CERT Resilience Management Model [69], ISO/IEC 27002:2022 [70], the NIST Privacy Framework [71], and the NIST Cybersecurity Framework [72]. Although TRICARE operation manuals provide clear guidance to providers related to background checks, patient privacy, and cyber hygiene [73], no single clearinghouse provides ethics training, employee background checks, security information and event management solutions, and SRAs for their civilian providers.

By publishing NPI numbers alongside provider contact details, the DHA could reduce the likelihood of fraudulent claims and improper payments. Whereas NPI numbers are permanent, providers may change or add business names, last names, locations, specialties, and state registrations. Furthermore, no free web-based search tool continuously gathers data from all state, federal, and licensing board databases using a combination of name and address spellings.

### Limitations

Our study was conducted under strict resource and time constraints. Between June 2022 and June 2023, we filed 4 Freedom of Information Act (FOIA) requests for (1) NPI numbers of TRICARE civilian providers, (2) data on TRICARE provider roster use, (3) data on HIE Opt-Out requests, and (4) data related to HIPAA-mandated SRAs (also known as “Compliance Risk Assessments”) performed by MCSCs and TRICARE network providers. Although the DHA acknowledged each request, they fulfilled none as of the date of this publication. The DHA’s most recent FOIA disclosure was on July 8, 2015 [74].

This study identified two TRICARE West provider names with matches on the HIPAA breach list. The HHS Office of Civil Rights does not require medical practices to report privacy breaches that impact ≤499 patients. The vast majority of civilian health providers operate in small practices and do not conduct regular SRAs [21].

The DHA’s annual report states that 80% of the public-facing information of their provider directory is accurate [7]. It does not, however, indicate which portion of the provider directory is not accurate. The TRICARE West provider does not include middle initials with all names and a complete list of states in which their providers are licensed. To address these limitations, we spent time manually differentiating providers with common names. For example, our list of 2398 matches includes a common female name that appears 10 times. Each of these female providers lives in different zip codes or states, has different degrees, and practices completely unrelated types of medicine. In other words, expert review was necessary to ensure our final sample was valid and free of duplicates.

In April 2023, the DHA announced that they would replace Health Net Federal Services with TriWest Healthcare Alliance as the prime contractor for TRICARE West [75]. TriWest has made no announcements regarding the transition of TRICARE West’s provider referral website.

### Conclusion

Our study reveals that 6.08% of the provider names listed on TRICARE West’s provider directory match individuals listed on federal and state exclusion lists. To assist law enforcement, we provided all data and study materials to the DODIG on May 8, 2023, and the DHA-OIG on May 24, 2023, in the form of a whistleblower complaint. To triage future threats associated with excluded and sanctioned provider names, we proposed a zero trust–based Insider Threat Management Model for TRICARE beneficiaries with security clearances. In future studies, we intend to compare TRICARE East, Medicare, the Children’s Health Insurance Program, and Substance Abuse and Mental Health Services Administration provider rosters against a broader spectrum of exclusion, sanction, and violation categories (ie, the Federal Sex Offender Registry). We also intend to develop products and interventions to automate background checks, protect patient privacy, and educate health care administrators about insider threats.

## Abbreviations

CRNA: certified registered nurse anesthetist
CSL: Consolidated Screening List
DFAS: Defense Finance and Accounting Services
DHA: Defense Health Agency
DO: doctor of osteopathic medicine
DODIG: Department of Defense’s Office of Inspector General
FAR: Federal Acquisition Regulation
FBI: Federal Bureau of Investigation
FDA: Food and Drug Administration
FOIA: Freedom of Information Act
FQHC: Federally Qualified Health Center
FUG: list of fugitives
GSA: General Services Administration
HHS: Department of Health and Human Services
HIPAA: Health Insurance Portability and Accountability Act
HPSA: health provider shortage area
HRSA: Health Research Services Administration
ISDC: Interagency Suspension and Debarment Committee
JHIE: Joint Health Information Exchange
LCP: licensed clinical psychologist
LEIE: List of Excluded Individuals and Entities
MCSC: managed care support contractor
MD: doctor of medicine
MN: master of nursing
MTF: military treatment facility
NIST: National Institute of Standards and Technology
NPI: National Provider Identification
OIG: Office of Inspector General
PHI: personal health information
PI: Program Integrity
PII: personal identifying information
PT: physical therapist
RBT: registered behavior technician
SRA: security risk assessment
TFMR: Total Force Medical Readiness
